# Abstract of the Proceedings of the Buffalo Medical Association

**Published:** 1864-02

**Authors:** William Ring

**Affiliations:** Secretary pro tem.


ART. III.—*Abstract of the Proceedings of the Buffalo Medical Association.*

TUESDAY EVENING, Jan. 5, 1864.

Minutes of the last meeting read and adopted.

*Dr. Gay* read the following paper:

At a recent meeting of the Association, I made some remarks upon the treatment of fractures of the bones, and advocated what I was pleased to call the *non-compulsory* plan of treatment, a plan, which, while it does not dispense with suitable appliances, discards all complicated apparatus and the immoderate restraint and force usually employed by Surgeons.

I am reported as advocating, at the same time, measures, the most severe and arbitrary, and a policy styled the do-nothing policy.

I am at a loss to know how such misconstruction could have been given to my remarks, unless the spike of Malgaigne, which was advocated in one particular fracture *only*, was considered on the one hand severe and barbarous, and the use of adhesive straps and the ordinary appliances were considered, on the other hand, means so mild as to be classed under the head of the so called do-nothing policy.

My object is now to correct such misapprehension, if it exists. I stated then, and reiterate now the same views, viz: That when a fractured limb, after reduction, is placed in that position which insures to the patient least pain and least irritation of the muscles, and left in that position if possible, for a few days without the employment of any considerable force, the greater would be the tendency of the fractured bones, to assume and maintain their normal position. If this proposition be true, which I assume to be true, then as a logical sequence, the more you excite the muscles to contraction by the employment of much force in the onset, and the more irritation and pain you cause, so much the more abortive will be your efforts and the greater tendency of the fractured bones to assume an abnornal position.

I desire to say one word in regard to Malgaigne's Instrument. In the course of the discussion on a former occasion it was said that this instrument had never been used in this country. The absence of practical knowledge was no doubt the reason which led to its condemnation as an instrument of barbarity, and consequently unfit for use. Dr. Hamilton, in his work on Fractures and Dislocations, recommends it, and although he has not yet employed it, proposes to use it the first opportunity that offers in the treatment of the special fracture for which the instrument was designed, viz: Fracture of the upper extremity of the tibia. In an article published in the October number of the American Journal of the Medical Sciences for 1863, in reference to this instrument the author says: This is a new treatment, and the reason why it has not been adopted before this time is probably the repulsive appearance of the treatment to patients and friends. It is found by experience, however, that very little pain is occasioned by wearing for weeks a steel point applied with considerable force to the fragment to be held. * * This, as a treatment of fractures may be found to be less painful than apparently more comfortable modes of dressing. * * *

This is all I purpose saying by way of correction upon the subject of *extreme* measures and *non-compulsory* means of treatment.

In connection with this subject I desire to call attention to a new article which is destined soon to come into general use in surgery. I allude to

*corn chaff.* It may be procured at any of the grain Elevators. It may be used in all cases where bran is applicable. Dr. Enos, of Brooklyn first called my attention to it, since which I have used it.

It is light, elastic and porous, and when the dust is sifted from it is much superior to bran. The use of corn chaff has enabled me I think to add something of value to the contributions of surgery, especially that department of surgery which relates to fractures of the femur. For the treatment of this injury I have a method of my own, but for a part of which, am indebted to Dr. Swinburne, of Albany. I will not occupy your time in giving directions *how* to apply this method, nor the dressings, since one surgeon is presumed to be as competent nearly as another in applying any apparatus at hand. I can embrace all I wish to say by simply naming the different parts of the apparatus.

These consist of the perineal and crural bands, scultetus bandage, long corn chaff cushion, for the leg to rest upon, short thigh splints, and straps to fasten them. The counter-extending band is made long, stuffed at its middle with the chaff, and the ends fastened to the head board of the cot which the patient is supposed to rest upon, the extending band, to which is attached a weight, rests over the foot board. The pulley is dispensed with, as it is believed the extension is more steady and uniform without, than with it.

I can hardly see how the bandage of scultetus can be dispensed with, since its application is so simple, and since nothing else will so perfectly control, by uniform pressure around the thigh, the contraction of the muscles. The side splints padded with chaff are strapped on and buckled tightly. This method discards altogether the long side splints as a bungling and unsightly contrivance, and as useless as bungling.

After the first dressing the whole may be removed, the thigh exposed, a soiled scultetus bandage exchanged for a clean one, without the least motion of the limb or pain or inconvenience to the patient. By this method I am at this present time treating an oblique fracture of the femur; the fracture occurring at the junction of the upper with the middle third of the shaft, in a young and muscular patient, and now, eleven weeks after the accident, by the most accurate measurement, the shortening does not exceed one quarter of an inch; the patient, during the whole period of treatment, having been much more comfortable than when burdened with more complicated apparatus.

*Dr. Peters* presented the following:

I was invited, together with Dr. Ross, by Dr. James P. White to accompany him, December 12th, 1863, to Niagara county to witness the removal of a tumor, supposed to be epithelial cancer, from the labium, and by his courtesy I am permitted to report it here as *apropos* to the discussion had at our last meeting. Found the patient, Mrs. G., a rather tall, spare lady, of 47 years, good general health, and presenting no indications of any unhealthy cachexia. The tumor was first observed about fifteen months since and gradually increased in size until about three months ago, when some escharotic application was made to it, since which time it has been ulcerated and at present discharges a yellow unhealthy pus, of a very unpleasant odor. She has suffered much pain from it and been obliged to take considerable quantities of opium.

The tumor is about the bulk of a hen's egg, though more elongated, reaching from the clitoris to the posterior commissure of the labia, and involving the whole right labium. The orifice of ulceration is of a size to admit one's finger, and the whole inside of the tumor seems to be fast disappearing by the process of ulceration. Dr. White proceeded to operate, assisted by the attending physician, Dr. Wilkins of Middleport, Dr. Ross, and myself. Partial anaesthesia having been produced by means of chloroform, the tumor was removed by a sort of oval shaped incision, and the edges brought together by means of one silk and seven silver sutures. Three arteries were ligated, and the loss of blood, though of course considerable, was not at all excessive. The patient bore the operation well, and being put to bed with her limbs fastened together to prevent any strain upon the sutures from her movements, a full dose of opium was given her, and we left her. Letters received from Dr. Wilkins to a recent date state union to have taken place, the sutures being removed, and the patient walking about.

A careful microscopical examination of the tumor was made by Dr. Mason and the caudate cell containing enlarged nuclei and nucleoli, said to be characteristic of epithelial cancer, was found in abundance. An examination of the base and sides of the mass failed to show any evidence of disease, so that it was concluded that the morbid growth had all been removed.

The first, most natural inquiry in all cases of operation on a malignant growth is, "Will it return?" for if it do the life of the patient is sacrificed. This is a question easy to ask, but to answer it requires not only a complete knowledge of all the facts in regard to the particular case in question, but a thorough discussion of the pathology of such tumors in general. Can we or can we not cure cancer? Is medical treatment ever of any use in its removal? We cannot answer these questions until we know what cancer is. If it be something heterologous, extraneous to the body, which fastens itself upon the living tissues, and like some foul fiend,

"*Monstrum horrendum, informe, ingens,*"

goes eating its way to the vitals of its victim, then we should hardly expect either the remedies of the physician, or even the knife of the surgeon, to be of much avail against it.

This has been substantially the view held of it in all ages, and it is undoubtedly to some such idea that it owes it name—Cancer. But we are every day learning that neither the antiquity of an idea nor its novelty necessarily proves its truth. If however, on the other hand, what we term cancer be, not an entity, but a pathological process; not the development of a new growth, but a change in the one already existing; then we should suppose *a priori*, that there could be found some stage in the process at which medication might be available. And this I believe to be the correct idea. To Robin, the microscopist, more than to any other man, the world of medicine is probably indebted for the elucidation of this question.

Starting with the philosophical idea that to know disease we must first know health, this observer first set himself to learn the different phases of normal development of the various tissues of the body, and then armed with this knowledge, boldly proceeded to the investigation of the different kinds of cancer.

The result of this investigation has been to show that what is known as cancer has no real existence, and that what is termed "cancer cell" is only the normal cell of the tissue affected, whose development has either been arrested previous to its arrival at maturity, or has gone on to a stage of degeneration beyond that point. And that we are to attribute the malignancy of a tumor, not to the irruption of some heterologous element into the body, but to a process set up in a tissue on account of some irritated or abnormal tendency of the constitution.

Precisely how this is done is of less consequence, so the fact be established; we are no more in the dark regarding it than we are regarding the progress of cancer under the old theory, or than we are regarding the production of pus. The views of Robin, set forth in the *Dictionnaires*

*de Medecine et Chirurgie*, etc., are much more strongly expressed than I have here indicated, and that I may not be thought to exaggerate I translate, (from the art. *Heteromorphe*) a few sentences expressive of his views.

"But there is not any more a heteromorphous generation, or a pathological heteroplasty, than there are heteromorphous or heteroplastic substances, elements or tissues. Their existence has been supposed, in default of knowing the facts relative to the generation of the elements, &c.; in default of knowing to what degree their aberrations of development can be extended comparatively to the normal phases of their evolution; in default of being able to connect their different morbid states to the normal states from which they are derived. Thus the words cancerous, schirrous, carcinomatous cells, or their analogues, consequently designate only a *state*, an accidental or morbid phase of evolution, most often of epitheliums, and sometimes of embryoplastic nuclei. But they do not designate a heteromorphous, or determinate and distinct species of element, or of tissue, a species which cannot be connected by its structure, its evolution, and its other properties to the natural tissues."

From these facts it seems to me that a tumor cannot be said to be necessarily malignant *ab initio*, but on the other hand, that any tumor may undergo this process of cell degeneration and exhibit the characteristics of malignancy. The practical application of this would seem to be that some remedy may yet be discovered which shall serve to check this process, and that we should not neglect medical treatment in the early stages of any tumor since any tissue may undergo the malignant degeneration.

The time having arrived for the consideration of the "Special Subject" designated for discussion—*Dr. Rochester* rising in response to the call of the presiding officer said, that in proposing Delirium Tremens, at a previous meeting, he did so, with the express avowal and understanding, that he was not to lead off in the debate, his object being to gain rather than to impart information; but as no one else had volunteered to present the matter, and as it seemed expected of him, he would not decline to bring it forward. Without indulging in any prefatory remarks as to the prevalence or pathology of the disorder, he recognized two distinct forms of the affection, which were perhaps susceptible of many sub-divisions. *First*, a wild frenzy, attended with heat of skin, increased arterial action, flushed face, and often irritable stomach, with gastric tenderness and unappeasable thirst, especially for ardent spirits. This was commonly the result of a protracted debauch, the effect of direct and continued alcoholic gastro-cerebral stimulation, and was often manifested when there was no cessation of inordinate dram drinking. This was the veritable *mania a potu.* The *Second* fo·m was a condition of extreme nervous excitement, with physical prostration, sleeplessness, delusions, and inappetency, attended with coolness or moisture of the surface, with a moist, tremulous and slightly furred tongue, with often pallor of the countenance, alternating with flushing—with feebleness of pulse, and without urgent thirst, but with great longing for alcohol. This is usually the result of forced or voluntary abstinence from the habitual excessive use of ardent spirits, and is the true *delirium tremens.*—Between these two forms there are many gradations, and as each particular instance approximates more nearly to the first or second form, there should be a corresponding difference in the treatment adopted. This distinction is not always drawn, and hence perhaps the conflicting opinions of medical men upon the subject. They are both instances of *oino-mania*, but widely different in many essential particulars. The first form is a gastritis with cerebral complication, which latter is probably mere functional excitement. This variety is "cured" by treament appropriate for gastritis, by counter-irritation over the epigastrium, by swallowing ice, by cold applications to the head, by cathartics acting as derivatives, and by being judiciously let alone. The second form we know less of as respects its pathology; it is the more common, and the more fatal disorder—it is that to which, especially, the inquiry this evening is directed. One tells us, vaguely, that it is "Alcoholismus," a vinous saturation of the system, and especially of the brain, and that when this is not maintained either by continued supply or by failure of effect, the result is, exhausting depression. Another defines it as Arachnitis. Another regards the alcoholic poison as analogous to uræmia; and still another tells us that the whole difficulty is gastric, and that sub-acute gastritis, softening of the mucous membrane of the stomach, and consequent cerebral sympathy; with the resulting arrest of the digestive and alimental functions, disposes of, and explains the whole subject.

Dr. Rochester, leaving this vexed question to be discussed by others, then proceeded to the consideration of treatment. This unfortunately was rather a matter of empirical experience, than the result of any well established pathological reasoning. It had been his lot to have seen a vast deal of delirium tremens from his connection for the last fifteen years with the hospitals in this and New York city, and the indications, to his mind, were clearly, to support the strength and at the same time allay the excitement of the patients. This can generally be accomplished by the administration of concentrated nourishment, (as beef essence,) by opium, and by stimulants—they are mentioned in the order of their relative importance. Dr. Rochester had employed and had seen used various methods of treatment, as, stimulation with carbonate of ammonia, the use of the shower bath, the inhalation of chloroform, confinement in dark and in light rooms, the administration in large doses of anti-spasmodics, solely—or narcotics solely as assafœtida, valerian, Hoffman's anodyne, conium, hyoscyamus, belladonna, etc.; he had never given digitalis in the monstrous and dangerous doses recently fashionable, and did not think that practice would be of very long continuance. It is objected to the use of opium, that from an overdose, or from the cumulative effects of repeated doses, fatal narcotism is often induced. This may occasionally be true, but in nine cases out of ten, the fatal coma is the result of asthenia, and this condition is often seen when no opium has been administered. In advocating the nutrient, opiate and stimulating plan of treatment, with the supplementary employment of bitter infusions Dr. Rochester was fully aware of its occasional failure, but thought it, on the whole, the safest and most successful course.

Dr. Rochester desires us to add to the above report of his remarks, the suggestion of the probable success of the hypodermic injection of morphine, as prompt and decided in its action, and certainly not liable to cumulative effects, from any temporary gastric insensibility.

*Dr. White* said that the views so ably advanced by Dr. Rochester were taught him by his preceptors more than thirty years ago, Dr. Marshall, and Dr. Trowbridge, whose portrait is before us. As to the pathology of this disease we know little or nothing. During the period of his practice he had tried a great variety of remedies—antimony, anti-spasmodics, chloroform, etc., etc., and after each deviation from the original plan, had returned to it with renewed confidence. This consists, in brief, in liberal use of stimulants, short of intoxication, opium to obtain sleep, short of narcotism, nutriment, counter-irritation, moderate restraint under the care of a good nurse. These were the sentiments with which he commenced practice thirty years ago, and the same rules of practice govern him now. There had been little or no progress made in the pathology and treatment of this disease during the entire period. Would notice a remedy mentioned by a British practitioner with high encomiums, ℥ii of red pepper in O j water, ℥ii, every half hour. Chloroform he has given, but it produced convulsions so that patient was in danger of death, and he had given up the practice.

*Dr. Strong* said that although the subject, so far as regards its nature, conditions and treatment has been discussed somewhat extensively, it still seemed to him that some points had been overlooked. For instance, in the more common form of the disease to which we are called, and which has now been mostly under discussion, whilst I accord to the correctness mainly of the principles and practice contended for by Drs. White and Rochester, viz: Opium to obviate insomnia and restlessness, with as free alimentation as possible, and alcoholic stimulus, q. s. to obviate exhaustion. Yet I have thought that it is too often forgotten when called to treat a fierce attack of this form of the disease that we have before us, primarily and essentially a case of gastritis—of a peculiar character, and from a peculiar cause, it is true, but still gastritis to all intents and purposes. And this being the condition, I have been in the habit in cases that did not readily yield, of superadding to the above, *emp. epis.* over the stomach, with agreeable results. And although I have never had occasion to adopt it, from the fact that other means have proved efficacious, yet by parity of reasoning—the stomach being the seat primarily of the disease—and that disease being more or less acute inflammation of one or more of its coats, I should regard it as good practice to *leech* or *wet-cup* over the same locality.

Reference has been made by Dr. White to the administration of cayenne. I have prescribed it in a case or two, though not, as by the gentleman alluded to by Dr. W., as a main reliance, excluding opium, but conjoined with that agent, and after it, alone, had failed of controlling the disease. Thus superadded, cayenne had acted admirably, by way apparently of increasing the susceptibility of the stomach to the effects of opium, and intensifying its powers. Viewed theoretically it may seem to some as acting on the principle of "*similia similiabus*," etc., or of adding fuel to the flame. Yet practically no such result will be verified, and I feel sure that gentlemen adopting it, in the case and for the purpose specified, will be pleased with the intensified potency which it imparts to our sheet-anchor, *opium.*

*Dr. Wyckoff* had, in the early part of his practice, used antimony extensively, but for the last few years had abandoned its use; relied now principally upon opium. In some instances after using opium largely without inducing sleep, administered chloroform by inhalations, and found that frequently after a patient became quiet from its effects he would sleep for hours under the influence of the opium previously taken.

*Dr. Lockwood* referred to a case which had greatly interested him; the patient had had delrium every year, or oftener, for a period of eleven years, and had recovered mainly, after a few of the first attacks, under the treatment of his wife, who gave saline cathartics and nutritious food; under this management, with confinement to the house, he soon became rational. He also remarked, that though various improvements in the treatment of delirium had been suggested and urged, there had really nothing important been gained for the last quarter of a century; the treatment then more generally adopted, was still regarded as most valuable. Dr. Coats of Philadelphia, had written a paper in 1827, and described a plan of treatment in this disease which had never been improved upon.

*Dr. Cronyn* observed that it had been his lot to have seen many cases of delirium tremens. Thought that the great difference of opinion in regard to the pathology and treatment of the disease arose from want of due discrimination of the form of the affection present in any one or more cases. Considered the division made by Mr. Solly of London, most conducive to correct and successful practice. Quoted several authors, as Dr. Locock, Higginbotham, Graves, etc., and their opinions, and mentioned some instances as they occurred in his practice.

*Voted* to adjourn.

WILLIAM RING,
*Secretary pro tem.*

